# Acupuncture for gouty arthritis: A PRISMA-compliant protocol for a systematic review and meta-analysis of randomized controlled trials

**DOI:** 10.1097/MD.0000000000023527

**Published:** 2020-12-04

**Authors:** Gamseong Lee, Foo Young Cho, Bonhyuk Goo, Yeon-Cheol Park

**Affiliations:** a Department of Clinical Korean Medicine, Graduate School, Kyung Hee University, 26, Kyungheedae-ro, Dongdaemun-gu, Seoul 02447; b Department of Acupuncture & Moxibustion, Kyung Hee University Hospital at Gangdong, 892, Dongnam-ro, Gangdong-gu, Seoul 05278; c Department of Acupuncture & Moxibustion, College of Korean Medicine, Kyung Hee University, 26, Kyungheedae-ro, Dongdaemun-gu, Seoul 02447, Republic of Korea.

**Keywords:** acupotomy, acupuncture, electro-acupuncture, fire-needling acupuncture, gouty arthritis, systematic review

## Abstract

**Backgroud:**

Patients with gouty arthritis suffer from intermittent attacks of pain, chronic inflammation, and joint damage. Acupuncture has been used in East Asian countries for centuries to treat various diseases, and several clinical studies have reported that acupuncture has beneficial effects on gouty arthritis. This study aims to evaluate the effect of acupuncture in patients with gouty arthritis by conducting a systematic review and meta-analysis.

**Methods:**

A comprehensive search of 8 electronic databases will be performed, including MEDLINE, Embase, the Cochrane Central Register of Controlled Trials, 4 Korean databases (KoreaMed, Korean Studies Information Service System, Research Information Service System, and Oriental Medicine Advanced Searching Integrated System), and 1 Chinese database (China National Knowledge Infrastructure). Only randomized controlled trials comparing acupuncture to conventional treatment and acupuncture with conventional treatment to conventional treatment alone for gouty arthritis will be included. Pain intensity will be considered the primary outcome. The secondary outcomes will include the pain relief duration, total effective rate, blood uric acid level, inflammatory markers, and incidence of adverse events. Two independent researchers will perform the study selection, data extraction, and quality assessment. The methodological quality of the individual included studies will be assessed using the Cochrane risk of bias tool. In the meta-analysis, for dichotomous and continuous data, risk ratios and standardized mean differences, respectively, will be estimated in addition to 95% confidence intervals.

**Results:**

: This systematic review will evaluate the effect of acupuncture treatment for patients with gouty arthritis with respect to clinical symptoms, laboratory indicators, and safety.

**Conclusion:**

: Our findings will help to establish the evidence of acupuncture to treat gouty arthritis.

**Registration number:**

PROSPERO CRD42020169668.

## 1 Introduction

Gouty arthritis is a severe, often debilitating type of inflammatory arthritis caused by the deposition of monosodium urate crystals in the joint space, periarticular structures, and soft tissues. This produces a pronounced inflammatory response that patient experiences as pain.^[1]^ Gouty arthritis affects approximately 1% to 2% of adults in the United Kingdom, mainland China, Polynesia, and Africa; 3% of those in Hong Kong; and more than 3% of those in the United States.^[2–5]^ Although the disease is well understood and effective treatment options are available, gouty arthritis is often poorly managed by both primary care physicians and rheumatologists.^[1]^

The current therapeutic options available for relieving pain in patients with gouty arthritis are limited to colchicine, non-steroidal anti-inflammatory drugs (NSAIDs), and corticosteroids. However, the use of these drugs often leads to severe adverse effects such as adverse gastrointestinal effects, chronic renal insufficiency, dysphoria, and immune suppression.^[6]^ Therefore, recent research has focused on the potential use of complementary and alternative medicine to treat gouty arthritis without significant adverse effects. Acupuncture has received particular attention because it is popular with patients, especially for pain-related conditions.^[7]^

Generally, acupuncture stimulation is thought to activate receptors and nerve fibers located deep within acupuncture points. This promotes cutaneous and deep tissue injury, which may be accompanied by local inflammatory responses and the excitation of various nociceptors.^[8,9]^ Endogenous opioid peptides and monoamines such as serotonin and norepinephrine are thought to mediate the analgesic effects of acupuncture.^[10–12]^ The analgesic effects of acupuncture support the possibility that acupuncture could be used as an alternative treatment for gouty arthritis.

Two systematic review studies were conducted in 2013 and 2016 to assess the clinical benefits of acupuncture therapy for gouty arthritis.^[13,14]^ However, only a small number of studies were included in this review, and those that were included were deemed to be of low quality, so the effect of acupuncture on gouty arthritis was not clearly established. Furthermore, in our preliminary search of the electronic databases, we identified that the evidence base that could be used to supplement this previous review covers various types of acupuncture such as manual acupuncture, electro-acupuncture, acupotomy, and fire-needling acupuncture.

This study aims to establish comprehensive evidence about the effect of acupuncture for patients with gouty arthritis. Therefore, we will conduct a systematic review and meta-analysis of randomized controlled trials (RCTs) to compare acupuncture versus conventional treatment and acupuncture with conventional treatment versus conventional treatment alone.

## 2 Methods

### 2.1 Study registration

This protocol was registered with the PROSPERO (registry number: CRD42020169668; http://www.crd.york.ac.uk/PROSPERO) and was designed according to the preferred reporting items for systematic reviews and meta-analysis protocols (PRISMA-P) 2015 Statement.^[15]^

### 2.2 Eligibility criteria

#### 2.2.1 Types of studies

To evaluate the effect of acupuncture for patients with gouty arthritis, only RCTs published in English, Korean, and Chinese will be included. Non-RCTs, quasi-RCTs, animal studies, case series, case reports, uncontrolled trials, and laboratory studies will be excluded.

#### 2.2.2 Types of patients

Patients diagnosed with gouty arthritis will be included. There will be no limitations imposed on the disease severity, age, gender, race, and education status of the patients.

#### 2.2.3 Types of interventions

Acupuncture as an experimental intervention is defined as the penetrating stimulation of acupoints by needles. Many types of acupuncture exist, such as manual acupuncture, electro-acupuncture, acupotomy, and fire-needling acupuncture. Studies in which the experimental group was treated with acupuncture or acupuncture with conventional treatment will be included, regardless of the number, duration, and frequency of treatment sessions. Studies involving other types of stimulation such as transcutaneous electrical nerve stimulation, pharmacopuncture, or acupressure will be excluded.

Studies in which the control group was treated with conventional treatments such as colchicine, NSAIDs, and corticosteroids will be included. RCTs that compared 2 different types of acupuncture or compared acupuncture to other traditional interventions such as moxibustion will be excluded.

In summary, the following comparisons will be addressed:

1. Acupuncture versus conventional treatment.
2. Acupuncture with conventional treatment versus conventional treatment alone.

### 2.3 Types of outcome measures

#### 2.3.1.1 Primary outcomes

The primary outcome will be pain intensity, which will be assessed using a pain score such as visual analogue scale or numeral rating scale.

#### 2.3.1.2 Secondary outcomes

The secondary outcomes will include:

1. Pain relief duration,
2. Total effective rate,
3. Blood uric acid level,
4. Inflammatory markers such as erythrocyte sedimentation rate and C-reactive protein, and
5. Incidence of adverse events.

### 2.4 Information sources and search strategy

We will retrieve literature published from January 1, 2000 to June 31, 2020 from the following 8 electronic databases, including MEDLINE, Embase, the Cochrane Central Register of Controlled Trials, 4 Korean medical databases (KoreaMed, Korean Studies Information Service System, Research Information Service System, and Oriental Medicine Advanced Searching Integrated System), and 1 Chinese database (China National Knowledge Infrastructure). Table 1 shows the strategy which will be used to search in MEDLINE via PubMed. Since all the databases searched possess their own subject headings, each database will be searched independently.

**Table 1:** Search strategy for the MEDLINE via PubMed.

((“gout”[MeSH Terms] OR “gout”[All Fields] OR (“Arthritis”[MeSH Terms] OR “Arthritis”[All Fields] OR “arthritides”[All Fields] OR “polyarthritides”[All Fields]) OR “tophus”[All Fields] OR ((“Gouty”[Title/Abstract] OR “gout^∗^”[Title/Abstract] OR “tophus”[Title/Abstract] OR “tophi”[Title/Abstract]) AND (“Arthritis”[Title/Abstract] OR “acute”[Title/Abstract]))) AND (“Acupuncture”[Title/Abstract] OR “acupuncture therapy”[Title/Abstract] OR “electro acupuncture”[Title/Abstract] OR “electrical acupuncture”[Title/Abstract] OR “fire acupuncture”[Title/Abstract] OR “needle knife”[Title/Abstract] OR “acupuncture point”[Title/Abstract] OR “chinese acupuncture”[Title/Abstract] OR “electroacupuncture”[Title/Abstract] OR “needleknife”[Title/Abstract] OR “Acupotomy”[Title/Abstract]) AND (“randomized controlled trial^∗^”[Title/Abstract] OR “clinical trial^∗^”[Title/Abstract] OR “random^∗^”[Title/Abstract])) NOT (“animals”[MeSH Terms] NOT “humans”[MeSH Terms])

### 2.5 Data collection and analysis

#### 2.5.1 Selection process

**Figure 1:**
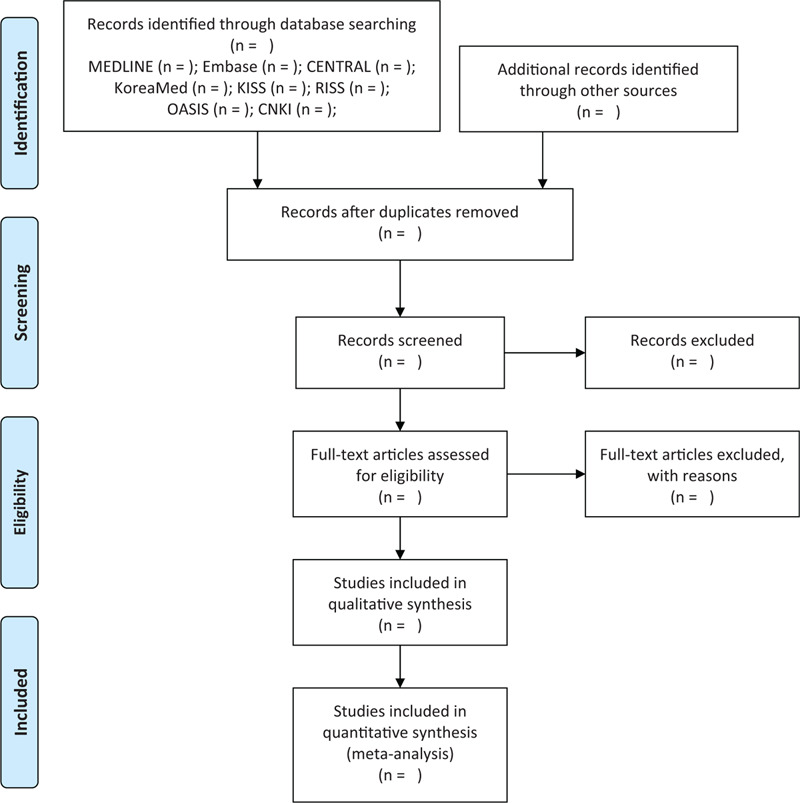
PRISMA flow diagram. CENTRAL = Cochrane Central Register of Controlled Trials, CNKI = China National Knowledge Infrastructure, KISS = Korean Studies Information Service System, OASIS = Oriental Medicine Advanced Searching Integrated System, PRISMA = Preferred Reporting Items for Systematic Reviews and Meta-Analyses, RISS = Research Information Service System.

Two reviewers (GL and BG) will independently screen the titles and abstracts of the retrieved articles to exclude any obviously irrelevant articles. Moreover, any duplicate studies will also be excluded. The full texts of the remaining articles will be downloaded and their suitability for inclusion in the review assessed using predetermined criteria. Disagreements between the 2 reviewers will be resolved by discussion. If the 2 reviewers do not reach an agreement, a third reviewer (Y-CP) will make the final decision. The study selection process will be recorded in the form of a PRISMA flow diagram (Fig. 1).^[16]^

#### 2.5.2 Data management and extraction

Having identified all of the studies which meet the inclusion criteria, a standard data collection form will be created before data extraction. Two reviewers (GL and BG) will independently extract data from the selected studies and complete the data collection form for each. Any discrepancies or uncertainties will be resolved through discussion with a senior reviewer (Y-CP). If information is missing from a study, we will contact the corresponding author for clarification. All data will be cross-checked and transferred into Review Manager. The following data will be extracted:

1. General information: research identifier, publication year, article title, first author, corresponding author, contact information, journal name, and country.
2. Study methods: study design, sample size, randomization method, allocation concealment, blinding, incomplete or selecting report, and other sources of bias.
3. Participants: inclusion and exclusion criteria, age, sex, race, onset, diagnostic criteria for gouty arthritis.
4. Interventions: type of acupuncture/control intervention, treatment details, treatment duration, and treatment frequency.
5. Outcomes: primary, secondary, and safety outcomes as described above.

#### 2.5.3 Risk of bias in individual studies

The risk of bias in the included studies will be assessed independently by 2 reviewers (GL and BG) using the Cochrane risk of bias tool. The following domains will be assessed: random sequence generation, allocation concealment, blinding of participants and personnel, blinding of outcome assessment, incomplete outcome data, selective outcome reporting, and other bias. Each domain will be assigned a low, unclear, or high risk of bias, and any discrepancies will be resolved by discussion between the 2 reviewers or by consulting a senior reviewer (Y-CP).

#### 2.5.4 Assessment of heterogeneity

We will assess heterogeneity by visually inspecting the forest plots to detect nonoverlapping confidence intervals (CIs) and by investigating the *χ*^2^ (a *P* value > .10 will be considered to indicate no heterogeneity) and *I*^2^ statistics. An *I*^2^ value ≥ 75% will be considered to represent considerable heterogeneity. If considerable heterogeneity is identified, the possible causes will be explored by subgroup analyses.

#### 2.5.5 Data synthesis

We will use Review Manager 5.4 (The Cochrane Collaboration, London, United Kingdom) to perform this meta-analysis. For continuous data, the results will be presented as standardized mean differences with 95% CIs. For dichotomous outcomes, data will be expressed as risk ratios with 95% CIs. A random-effect or fixed-effect model will be used to calculate the pooled estimates of the effect size. If we are not be able to conduct a meta-analysis because of a lack of clinical studies or because of heterogeneity, we will present the effect size and 95% confidence interval (CI) of every outcome in each clinical trial and describe the significance of important results in Section 3. To summarize the findings of the meta-analysis and describe the strength of evidence, we will use the Grades of Recommendation, Assessment, Development, and Evaluation approach.^[17]^

#### 2.5.6 Subgroup analyses

We plan to carry out subgroup analyses if we identify substantial heterogeneity. Subgroup analyses will be conducted according to:

1. Type of acupuncture
2. Degree of severity
3. Type of conventional treatment in control group.

#### 2.5.7 Assessment of reporting biases

If the number of included studies is greater than 10, we will generate funnel plots to detect reporting biases and small-study effects.

## 3 Discussion

Gouty arthritis is a significant cause of morbidity, disability, lost work days, and high healthcare utilization due to intermittent attacks, chronic inflammation, and joint damage. Nowadays, anti-inflammatory and urate-lowering drugs are the most commonly used treatments for gouty arthritis. However, some patients with gouty arthritis desire non-pharmacological interventions which improve their symptoms without inducing adverse effects. Given its favorable side effect profile and low financial cost, acupuncture has been used to treat gouty arthritis for many years in East Asian countries.

Acupuncture stimulation triggers a sequence of events that include the release of neurotransmitters and endogenous opioid-like substances and the activation of c-Fos within the central nervous system.^[18]^ It can relieve pain associated with many acute and chronic pain conditions including gouty arthritis.

Generally, acupuncture treatment is used in the form of traditional manual acupuncture. However, various types of acupuncture techniques have been applied recently to increase the analgesic effect or obtain the additional effect than manual acupuncture. Electro-acupuncture is a technique that combines traditional acupuncture with electrical stimulation, and its analgesic effect on various types of pains through the mechanism related with endogenous opioids and descending inhibitory system was reported in many studies.^[19,20]^ Acupotomy is a special technique of acupuncture conducting minimally invasive surgery using a needle with a flat blade shape and causes natural opioid-mediated pain suppression by stimulating local alpha–delta nerve fibers.^[21,22]^ Fire-needling acupuncture is an acupuncture technique that inserts a heated acupuncture needle on the body to add cauterization effect, and it promotes microcirculation in the treated area through the regulation of cutaneous nerves, which is beneficial for the absorption of inflammatory metabolites.^[23]^ Because various types of acupuncture are used for the treatment of gouty arthritis based on these mechanisms, additional analyses of each technique are required along with analysis of the entire acupuncture treatment.

Although a review into the effects of acupuncture on gouty arthritis was previously conducted, this had a number of limitations and the clinical evidence has since been updated, so a comprehensive review is necessary to evaluate the latest evidence. We will conduct this systematic review and meta-analysis to evaluate the effect of acupuncture treatment for patients with gouty arthritis with respect to clinical symptoms, laboratory indicators, and safety. Our findings will help clinical practitioners and patients to establish treatment strategy for gouty arthritis, and researcher to seek the direction for further clinical research into the use of acupuncture to treat gouty arthritis.

## Author contributions

**Conceptualization:** Yeon-Cheol Park.

**Data curation:** Foo Young Cho.

**Formal analysis:** Gamseong Lee.

**Funding acquisition:** Yeon-Cheol Park.

**Investigation:** Gamseong Lee, Foo Young Cho, Bonhyuk Goo.

**Methodology:** Bonhyuk Goo.

**Project administration:** Bonhyuk Goo.

**Supervision:** Yeon-Cheol Park.

**Writing – original draft:** Gamseong Lee.

**Writing – review & editing:** Bonhyuk Goo, Yeon-Cheol Park.
